# Hemodynamic diagnostics of epicardial coronary stenoses: *in-vitro *experimental and computational study

**DOI:** 10.1186/1475-925X-7-24

**Published:** 2008-08-27

**Authors:** Rupak K Banerjee, Koustubh D Ashtekar, Tarek A Helmy, Mohamed A Effat, Lloyd H Back, Saeb F Khoury

**Affiliations:** 1 Department of Mechanical, Industrial and Nuclear Engineering, 601B Rhodes Hall, University of Cincinnati, Clifton Avenue, Cincinnati, OH, USA; 2 Department of Internal Med-Cardiology, MSB, University of Cincinnati, Cincinnati, OH, USA; 3 Jet Propulsion Laboratory, California Institute of Technology, Pasadena, CA, USA; 4 Department of Biomedical Engineering, 598 Rhodes Hall, PO Box 210072, Cincinnati OH, 45221 0072, USA

## Abstract

**Background:**

The severity of epicardial coronary stenosis can be assessed by invasive measurements of trans-stenotic pressure drop and flow. A pressure or flow sensor-tipped guidewire inserted across the coronary stenosis causes an overestimation in true trans-stenotic pressure drop and reduction in coronary flow. This may mask the true severity of coronary stenosis. In order to unmask the true severity of epicardial stenosis, we evaluate a diagnostic parameter, which is obtained from fundamental fluid dynamics principles. This experimental and numerical study focuses on the characterization of the diagnostic parameter, pressure drop coefficient, and also evaluates the pressure recovery downstream of stenoses.

**Methods:**

Three models of coronary stenosis namely, moderate, intermediate and severe stenosis, were manufactured and tested in the *in-vitro *set-up simulating the epicardial coronary network. The trans-stenotic pressure drop and flow distal to stenosis models were measured by non-invasive method, using external pressure and flow sensors, and by invasive method, following guidewire insertion across the stenosis. The viscous and momentum-change components of the pressure drop for various flow rates were evaluated from quadratic relation between pressure drop and flow. Finally, the pressure drop coefficient (CDP_e_) was calculated as the ratio of pressure drop and distal dynamic pressure. The pressure recovery factor (*η*) was calculated as the ratio of pressure recovery coefficient and the area blockage.

**Results:**

The mean pressure drop-flow characteristics before and during guidewire insertion indicated that increasing stenosis causes a shift in dominance from viscous pressure to momentum forces. However, for intermediate (~80%) area stenosis, which is between moderate (~65%) and severe (~90%) area stenoses, both losses were similar in magnitude. Therefore, guidewire insertion plays a critical role in evaluating the hemodynamic severity of coronary stenosis. More importantly, mean CDP_e _increased (17 ± 3.3 to 287 ± 52, n = 3, *p *< 0.01) and mean *η *decreased (0.54 ± 0.04 to 0.37 ± 0.05, *p *< 0.01) from moderate to severe stenosis during guidewire insertion.

**Conclusion:**

The wide range of CDP_e _is not affected that much by the presence of guidewire. CDP_e _can be used in clinical practice to evaluate the true severity of coronary stenosis due to its significant difference between values measured at moderate and severe stenoses.

## Background

In *current clinical practice*, the ischemic severity of epicardial coronary stenosis can be detected by functional and anatomical measurements. The anatomical or geometric measurements, performed by imaging methods (e.g., contrast angiography, angioscopy and intravascular ultrasound), may not reveal the true severity of coronary stenosis and may lead to inappropriate clinical decisions [1]. On the other hand, functional or hemodynamic measurements of mean pressure drop across the stenosis and distal coronary flow prove to be more useful for the long term success of balloon angioplasty with or without stent placement procedure [2]. Hence, the development of simple and effective clinical diagnostic methods utilizing these functional measurements has attracted the interest of many researchers. For this purpose, the intravascular clinical procedure is performed before the angioplasty by surgically placing a 2–2.3 mm diameter guide-catheter up to the coronary ostium. The 0.35 mm diameter pressure or Doppler flow sensor-tipped guidewire is advanced through this guide-catheter and placed across the stenosed coronary artery to measure distal pressure or flow [3]. Using this invasive pressure and flow measurement, various diagnostic parameters have been developed in current clinical practice e.g. coronary flow reserve (CFR: the ratio of hyperemic flow to the basal flow) [4], trans-stenotic pressure drop [5], fractional flow reserve (FFR: the ratio of distal recovered pressure to the aortic pressure at hyperemia) [6].

### Uncertainties in pressure-flow diagnostics

To distinguish epicardial stenosis severity from microvascular dysfunction, simultaneous measurements [7] of the coronary flow and pressure have been recently recommended [8,9]. For this purpose, both pressure and flow sensors are simultaneously inserted into the coronary vessel. However, the insertion of guidewires across the stenosis causes a "tight fit" in the lumen of the stenosed artery and is associated with additional flow reduction and an increase in mean pressure drop [2,5] that may result in an incorrect overestimation [10,11] of hemodynamic parameters [12] and may lead to improper selection of therapeutic procedures [13,14]. Possible risks and resulting consequences to the patients because of the uncertainties in pressure drop and flow measurements due to the size and number of guidewires inside the diseased vessel were recently reported. In earlier clinical study, clinicians observed that the diastolic pressure drop measured by a balloon catheter (1.98 mm size) was 34% more than that measured by a single guidewire (0.45 mm size) [14], typically used in present clinical settings. In a study of 65 patients, Verberne et al [11] reported a 25% increase in hyperemic pressure drop and a 20% reduction in hyperaemic flow with the use of two guidewires (separate pressure- and flow sensor- tips on different guidewires; a procedure not commonly used) instead of a newly developed single guidewire having both pressure and flow sensors on its tip. Further data analysis indicated 30%, 128% and 325% increase in the misdiagnosis (false positive cases) of the true ischemic severity of coronary stenosis due to wrong measurements of CFR, FFR and hyperemic stenosis resistance (hSRv: the ratio of hyperemic pressure drop and distal blood flow velocity), respectively [11]. The uncertainty in deciding the limiting values of CFR, FFR and pressure drop measured by different sizes of guidewires, has been experimentally [15] and numerically evaluated and discussed in detail in our previous studies [16,17].

We *hypothesized *that the hemodynamic parameter consisting of simultaneous measurements of pressure-flow integrated with anatomical details can overcome the disadvantages of conventional diagnostic parameters. Recently, our group has developed an alternative diagnostic parameter: Lesion flow coefficient (LFC: ratio of non-dimensional pressure drop at very high flow rate and at patho-physiological hyperemic flow rate) [17] to determine the true severity of epicardial coronary stenosis. Accordingly, in this study, we sought to investigate the behaviour of the non-dimensional hemodynamic or functional diagnostic parameter, pressure drop coefficient (CDP_e_), over a wide range of coronary flows simulated in an *in-vitro *experimental set up. The CDP_e _utilizes simultaneous pressure-flow measurement across the stenosis, while evaluating the downstream pressure recovery factor (*η*) that includes simultaneous pressure-flow measurement and anatomical details.

The *specific aims *of this study are: 1) to compare the pressure drop-flow relation for pulsatile flow in the presence and absence of single guidewire, 2) To evaluate the behaviour of hemodynamic parameter, CDP_e _while evaluating *η *for different degrees of stenoses under variable pressure-flow scenarios.

## Methods

### Formulation

#### Hemodynamic diagnostic parameter: pressure drop coefficient (CDP_e_)

We define the CDP_e _to be the ratio of mean trans-stenotic pressure drop, $\Delta \tilde{p}$, (superscript '~' indicates temporal average quantity) to the proximal dynamic pressure, i.e.,

(1) $$CDP_{e}=\Delta \tilde{p}/(0.5\times \rho \times {\tilde{\bar{u}}}_{e}^{2}).$$

where, *ρ *is blood density; ${\tilde{\bar{u}}}_{e}$ is spatial and temporal mean blood velocity in the proximal vessel (superscript '-' indicates a spatially average quantity). The $\Delta \tilde{p}$, in equation 1, is composed of viscous- ($\Delta {\tilde{p}}_{vis}=B\times \tilde{Q}$) and momentum-change ($\Delta {\tilde{p}}_{mom}=A\times {\tilde{Q}}^{2}$) related pressure losses, where A and B are momentum and viscous pressure loss coefficients, respectively. These pressure loss coefficients are the functions of flow rate (*Q*) and anatomical (geometrical) details of a stenosis [18]. Thus, the total pressure drop exhibits a quadratic relation with flow rate ($\Delta \tilde{p}=A\times {\tilde{Q}}^{2}+B\times \tilde{Q}$), which, in turn, influences CDP_e _for different size of stenotic geometries.

#### Pressure recovery factor (*η*)

Applying Bernoulli's equation to the stenotic geometry, pressure recovery coefficient (CPR) can be defined as the ratio of distal pressure recovery ($\Delta {\tilde{p}}_{r}$) to the dynamic pressure at the site of minimum vessel diameter ($0.5\times \rho \times {\tilde{\bar{u}}}_{m}^{2}$) i.e.

(2) $$CPR=\Delta {\tilde{p}}_{r}/(0.5\times \rho \times {\tilde{\bar{u}}}_{m}^{2})$$

where, ${\tilde{\bar{u}}}_{m}$ is spatial and temporal mean blood velocity at the minimal vessel area. Therefore, we define the *pressure recovery factor *(*η*) to be the ratio of experimentally measured CPR to the quantity *CPR*_∞_, that represents the pressure recovery coefficient at very high (limiting) Reynolds number (Re) flow, i.e.,

(3a) $$\eta =\frac{CPR}{CPR_{\infty }}=\frac{CPR}{\left(1-[1/\alpha^{2}]\right)}$$

where *CPR*_∞ _is expressed as

(3b) $$CPR_{\infty }=\frac{\Delta p_{r}}{0.5\rho .u_{m}^{2}}=1-{\left(\frac{u_{r}}{u_{m}}\right)}^{2}=1-{\left(\frac{A_{m}}{A_{r}}\right)}^{2}=1-\frac{1}{\alpha^{2}}$$

We define Δ*p*_*r *_= *p*_*r *_- *p*_*m*_and *α *is the ratio of the flow cross-sectional area of the distal vessel, A_r_, to the minimum vessel area at the site of stenosis, A_m_, prior to guidewire insertion.

(4) *α *= *u*_*m*_/*u*_*r *_= *A*_*r*_/*A*_*m *_= *A*_*e*_/*A*_*m*_

where, *A *is the cross-sectional area, subscript '*r*' represents the distal vessel; '*m*' represents the vessel at the site of stenosis with minimum flow area; '*e*' represents the proximal healthy vessel. For simplicity, it is assumed that *A*_*r *_= *A*_*e *_i.e. proximal and distal vessel diameters are the same. Similarly, we define *α*_*i *_to be the ratio of the flow cross-sectional area of the distal vessel (A_r_-A_i_) to the minimum vessel area at the site of stenosis (A_m_-A_i_), during guidewire insertion.

(5) *α*_*i *_= (*A*_*r *_- *A*_*i*_)/(*A*_*m *_- *A*_*i*_) = (*A*_*e *_- *A*_*i*_)/(*A*_*m *_- *A*_*i*_)

where, subscript '*i*' represents the guidewire. Further, the % occlusion of the vessel or % area stenosis is defined as follows:

(6) Percentage area stenosis = (*A*_*e *_- *A*_*m*_)/(*A*_*e*_)

Here, the *pressure recovery factor *(*η*) measures the ability of distal vessel to recover the pressure downstream to the stenosis [19] and have wide application in nozzle flow fluid dynamics.

### Experimental method

Simultaneously measured pressure and flow data for each stenosis test sections were compared with corresponding numerical calculations. The $\Delta {\tilde{p}}_{mom}$ and $\Delta {\tilde{p}}_{vis}$ were analysed for each stenosis section by comparing pressure-flow characteristics for both 'before' and 'during' guidewire insertion under pulsatile flow conditions. Finally, fluid dynamic parameters: overall pressure drop coefficient (CDP_e_) and the pressure recovery factor (*η*) were evaluated.

#### Coronary Stenosis Test Sections

**Figure 1:**
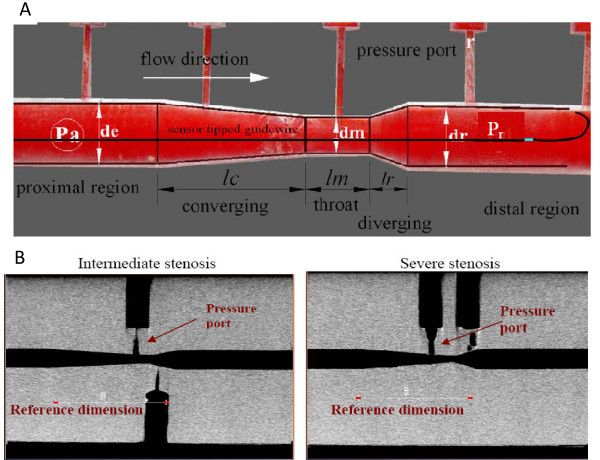
**Stenosis test section geometry**. **A **Moderate stenosis photograph with pressure ports. **B **– MicroCT images: intermediate stenosis, severe stenosis.

We created three model test sections to simulate different severity of epicardial focal coronary stenosis. These test sections are manufactured with optical grade Lexan material for following three severity conditions: moderate, intermediate and severe stenosis, having % area occlusion of 65%, 80% and 89%, respectively. Epicardial focal coronary stenosis consists of three distinct sections, converging or constricting, throat, and diverging section as shown in Fig. 1A, B[13,20]. The above manufactured test sections are as per the characteristic dimensions reported by Wilson et al. ([13], Table 1). To measure the axial pressure and time averaged pressure recovery (${\tilde{p}}_{r}$) in the distal part of the test section, 0.3 mm diameter radial pressure ports were drilled in the axial direction at an interval of ~5 mm as shown in Fig. 1. The proximal pressure (${\tilde{p}}_{a}$) was measured by pressure port located just proximal to the converging section while the throat pressure (${\tilde{p}}_{m}$) was measured by pressure port located at the middle of throat section. The mean trans-stenotic pressure drop in equation 1 can be calculated as: $\Delta \tilde{p}={\tilde{p}}_{a}-{\tilde{p}}_{r}$ and the mean distal recovered pressure in equation 2 can be calculated as:$\Delta {\tilde{p}}_{r}={\tilde{p}}_{r}-{\tilde{p}}_{m}$. Details of these models are reported in our recent publication [15]. Anatomy of stenoses have curvatures and transition from different sections of the stenosis are gradual; accordingly, three custom mill bits are used to sequentially drill, first, the throat section followed by the converging section from one end and diverging section from the other end.

**Table 1:** Dimensions for three stenotic test sections. All dimensions are measured with ImageJ software^®^

Dimensions (mm)	65% area (moderate) stenosis	80% area (intermediate) stenosis	89% area (severe) stenosis
proximal vessel diameter (*d*_e_)	2.95	2.95	2.96
length of converging section (*l*_c_)	6.96	6.35	6.28
throat minimal diameter (*d*_m_)	1.75	1.32	0.98
throat length (*l*_m_)	3.15	0.95	0.39
length of diverging section (*l*_r_)	1.79	1.62	1.59
distal vessel diameter (*d*_r_)	2.95	2.98	3.00

#### Experimental Setup

**Figure 2:**
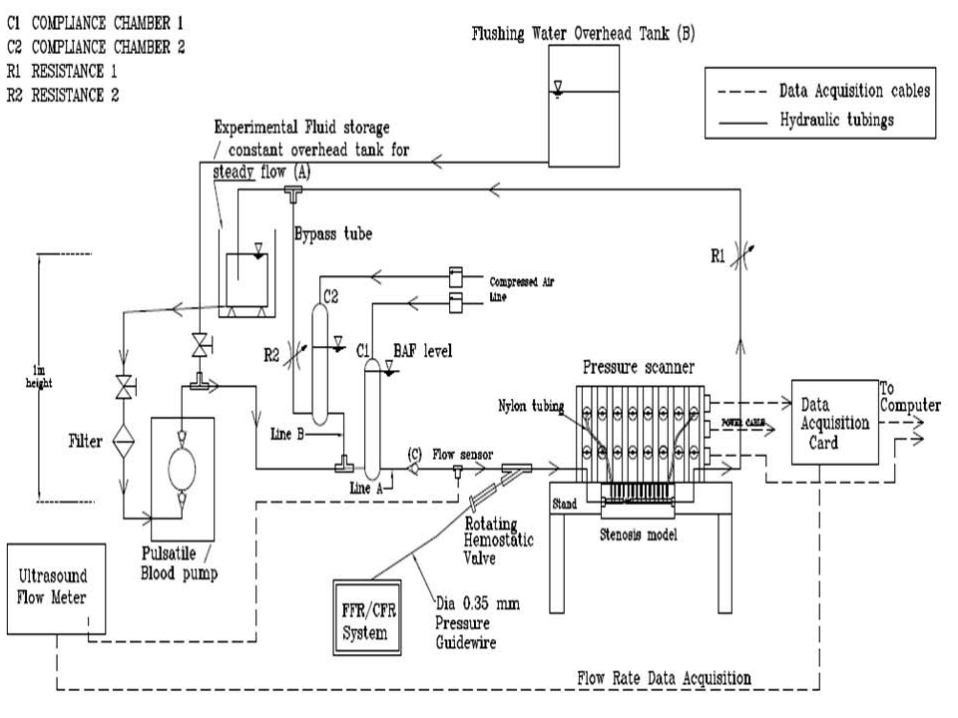
Experimental set-up.

**Figure 3:**
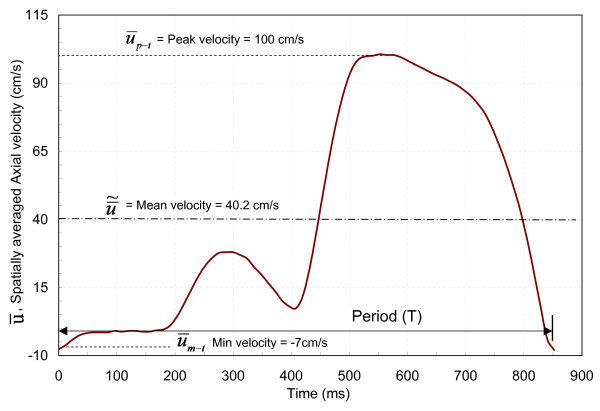
Experimental velocity pulse.

**Figure 4:**
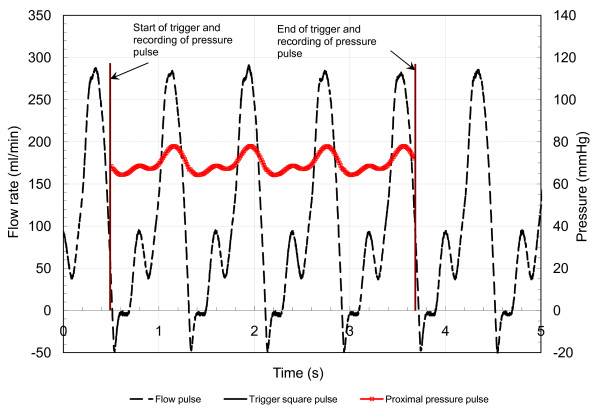
Simultaneous pressure-flow measurement. The square wave is used as a reference trigger for simultaneous measurement of pressure and flow. The reference starting time is the same for acquisition of both pressure and flow data.

The diastole-dominated coronary arterial flow waveform was generated by compliance-resistance method as explained in our previous *in-vitro *study [15]. The schematic diagram of experimental set up is shown in Fig. 2. The basic pulsatile flow waveform (T = 0.8 sec, $\tilde{\text{Q}}$ = 350 ml/min) was generated by the pulsatile pump (Harvard Apparatus, MA), which is similar to the aortic flow waveform. This flow was then bifurcated into two flow conduits: A and B in order to divert very high flow rate from the pulsatile pump. The compliances (C1 and C2), and resistances (R1 and R2) were connected and adjusted within the conduits to generate the physiological coronary arterial flow waveform (Fig. 3). The mean flow rate was increased from basal (~50 ml/min) to hyperemic flow (moderate: 180 ml/min, intermediate: 165 ml/min, severe: 115 ml/min) in four steps. The pulsatile pressure and flow were measured simultaneously with the help of a reference trigger square pulse. Figure 4 explains the method of simultaneous pressure-flow measurement. A typical left anterior descending (LAD) coronary flow pulse was reported by many researchers [21,22]. Generally, the systolic dominance in the aortic flow profile is reversed to diastolic dominance in the normal to moderately stenosed vessels. The experimentally obtained flow pulse (Fig. 3) was almost similar to the pulse velocity measured by a Doppler catheter in the LAD of patients undergoing angioplasty [21,22].

For reference steady flow experiments, gravity induced constant flow was supplied through the stenotic test section. The steady flow (*Q*) was increased from basal to hyperemic flow in four steps, while trans-stenotic pressure and flow were measured simultaneously. During each step of flow increment, the guidewire was inserted across the stenotic test section to measure trans-stenotic pressure and flow.

#### Pressure and Flow Measurements

The axial pressure was measured by a DSA3207 digital sensor array (Scanivalve corp., WA) with 12 ms time interval. The pulsatile flow was measured with an in-line Doppler flow sensor (Transonic Inc., NY) with a time interval of 1 ms. Simultaneous pressure-flow values were measured for the following two conditions:

1) Before guidewire insertion: This method can be considered as "non-invasive or patho-physiological" measurements, since pressure was measured without guidewire insertion across the stenotic models.

2) During guidewire insertion: This method can be considered as "invasive" measurements, since pressure was measured after guidewire insertion across the stenotic model. The guidewire was connected to the ComboMap system (Volcano Therapeutic, CA) for continuous pressure recording during its insertion across the stenotic models.

#### Blood Analog Fluid (BAF)

**Figure 5:**
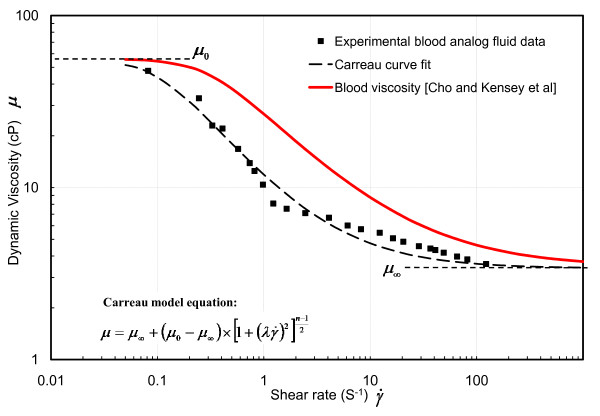
Non-Newtonian shear thinning viscosity: Carreau model.

The experiments were performed with a fluid exhibiting the shear thinning, non-Newtonian viscous property of blood. The BAF was prepared by mixing 65% water, 35% glycerine, and 0.02% Xanthum gum (by weight) [23]. The viscosity was measured by concentric cylinder viscometer (DV-II+ PRO Digital Viscometer, Brookfield, MA). The experimental data, showing shear-thinning behaviour of BAF, were fitted to Carreau model (Eq. in Fig 5), where the Carreau coefficients are as follows: zero shear rate viscosity, *μ*_0 _= 55 cP, infinite shear rate viscosity, *μ*_∞ _= 3.39 cP, time constant, *λ *= 9.56 s, and power index, *n *= 0.2. The comparison of BAF viscosity and real blood viscosity [24] is provided in Fig. 5. The density of BAF was measured as 1.05 g/cm^3^. The comparison of results between BAF and blood viscosities is discussed in Additional file 1.

### Numerical method

#### Geometry

**Figure 6:**
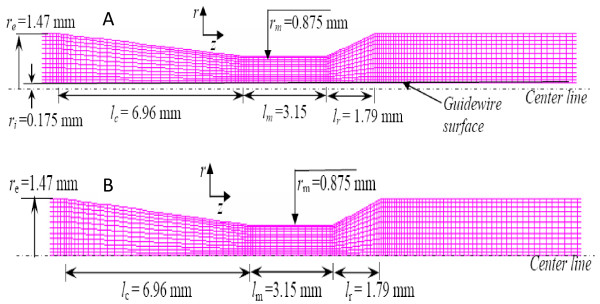
**Numerical models**. **A **– Numerical model for moderate stenosis (during guidewire insertion). **B **– Numerical model for moderate stenosis (before guidewire insertion).

The numerical calculations were performed based on the dimensions of *in-vitro *stenotic test sections as provided in Table 1. Two numerical models were generated for each stenotic test section; namely, before and during guidewire insertion. Mesh diagrams 'during' and 'before' guidewire insertion are shown in Fig. 6A and Fig. 6B. It is assumed that: (a) the arterial wall has a smooth, rigid and round concentric shape; (b) the stenosis geometry remains unchanged during basal and hyperemic flow due to failure of the flow-dependent dilation mechanisms in atherosclerotic coronary arteries; and (c) the rigid arterial wall has insignificant effect of pressure pulsation on its dimensions.

#### Boundary and Initial Conditions

The 'no slip' boundary condition, *u*_*i *_= 0, was specified on the arterial wall and on the guidewire wall (in case of guidewire inserted conditions). A stress free boundary condition (*σ*_*i *_= 0) was applied at the flow outlet. The symmetric boundary condition (*u*_*r *_= 0) was applied at the central axis in the case of 'before' guidewire insertion. The coronary flow waveform, *Q*(*t*), used in the computational analyses, was obtained from the corresponding *in-vitro *experimental data. The spatially averaged velocity along the vessel cross-section, $\bar{u}\left(t\right)$, needed for the computational analysis, was obtained from the mass balance equation: $\bar{u}\left(t\right)$ = *Q*(*t*)/*A*_*e*_. The $\bar{u}\left(t\right)$ with analogous *poiseuille flow *relation was applied at 3 cm (at the point where *l*/*d *= 10) proximal to the converging section of the numerical stenotic models in case of 'before' guidewire insertion case. Thus, the inlet boundary conditions are [25,26]:

(7a) $$u\left(r,t\right)=2\cdot \bar{u}\left(t\right)\cdot \left(1-{\left(\frac{r}{r_{e}}\right)}^{2}\right)$$

before guidewire insertion and

(7b) $$u\left(r,t\right)=2\cdot \bar{u}\left(t\right)\cdot \left[\left(1-{\left(\frac{r}{r_{e}}\right)}^{2}\right)\ln\left(\frac{r_{e}}{r_{i}}\right)+\left(1-{\left(\frac{r_{i}}{r_{e}}\right)}^{2}\right)\ln\left(\frac{r}{r_{e}}\right)\right]/\left[\left(1+{\left(\frac{r_{i}}{r_{e}}\right)}^{2}\right)\ln\left(\frac{r_{e}}{r_{i}}\right)-\left(1-{\left(\frac{r_{i}}{r_{e}}\right)}^{2}\right)\right]$$

during guidewire insertion for annular flow

Because of long entry length, the spatial velocity profiles at any instance of time adjusted to a profile that is consistent for non-Newtonian BAF fluid.

The temporal mean pressures were calculated along the arterial wall to find the mean pressure drop, $\Delta \tilde{p}={\tilde{p}}_{r}-{\tilde{p}}_{e}$ and mean pressure recovery, $\Delta {\tilde{p}}_{r}={\tilde{p}}_{r}-{\tilde{p}}_{m}$, where as ${\tilde{p}}_{e}$, ${\tilde{p}}_{r}$ and ${\tilde{p}}_{m}$ represent the mean pressure measured proximal, distal (at the site of pressure recovery) and at the throat region of the stenosis. The experimentally observed non-Newtonian viscosity and density of BAF were used for this calculation.

#### Solution Strategy

The unsteady problem was solved by finite element method utilizing the Galerkin scheme (FIDAP, ANSYS, Inc., NH). The pressure was discredited by mixed formulation and approximated as discontinuous across the element boundaries for this incompressible flow problem. The 2^nd ^order implicit trapezoidal time integration scheme was used to control local truncation error. A successive substitution type of fully coupled iterative solver was used to obtain the solution at each time step of this non-linear time-dependent problem. This method solves the linearized system of governing equations by direct Gaussian elimination approach. The convergence criteria for the velocity and residual vector were tightened to a value of 0.01% which is two orders of magnitude lower than the recommended value [27]. In order to optimize the convergence time at each time step, a relaxation factor was used (0.5). To achieve smooth converged results, a relatively small value of streamline upwinding (a value less than 0.45) that adds numerical diffusion along the flow direction was used to calculate primary flow variables (*u*). For these analyses, a Compaq Linux machine with dual processor (2.4 GHz, 1 GB RAM, 80 GB hard disk) was utilized so that CPU time for each time step was ~1.5 s.

#### Mesh refinement study

A series of meshes were created, with each one 20% higher in density than the previous one, in order to check the overall convergence accuracy of the numerical calculations [25-27]. When the improvement with the refined mesh was less than 1% in velocity vectors, wall shear stress, and pressure, the numerical calculation was considered to be converged. For pulsatile flow calculations, the convergence is not only dependent on the mesh resolution but also on the selection of the time step. Depending on the velocity pulse shape and stenosis geometry, the calculation time steps varied between 1 × 10^-4 ^to 1 × 10^-5 ^sec. These calculations were started from a time where the velocities were near zero in order to make the stiffness matrix well-balanced and stable. Considering the refinements in time-steps, and appropriate starting point of numerical calculations, one may not need much finer mesh for an unsteady calculation [25-27].

## Results

### Trans-stenotic mean axial pressure drop

The experimentally measured and numerically calculated temporal mean axial pressure data were analyzed to determine the overall mean trans-stenotic pressure drop ($\Delta \tilde{p}$), mean distal pressure recovery ($\Delta {\tilde{p}}_{r}$). The $\Delta {\tilde{p}}_{vis}$ and $\Delta {\tilde{p}}_{mom}$ were quantified based on the experimentally measured mean pressure drop and mean flow rate values having quadratic relation.

#### Moderate (65% area) stenosis

**Figure 7:**
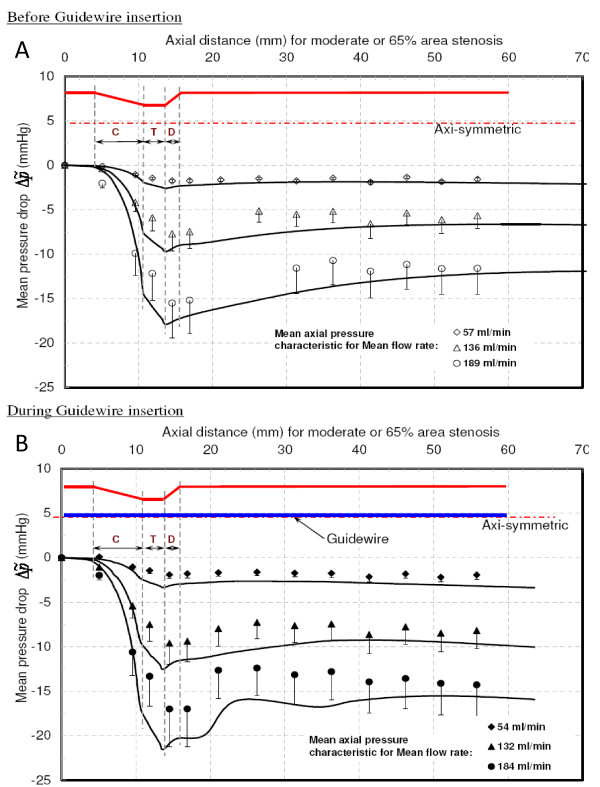
**Mean pressure drop vs. Axial distance for moderate stenosis**. **A **– Before guidewire Insertion. **B **– During guidewire Insertion. C = converging region; T = throat region; D = diverging region. . . . .: Experimental data points. : Numerical results.

Figure 7A shows the trans-stenotic mean axial pressure as a function of axial distance before guidewire insertion. The values of overall pressure drop ($\Delta \tilde{p}$) and distal pressure recovery ($\Delta {\tilde{p}}_{r}$) for mean flow rates of 57, 136, and 189 ml/min are summarised in Table 2. The distal pressure recovery considerably reduced the overall pressure drop. The CPR increased from 0.42 to 0.52 as Reynolds number (Re_e_) increased from 125 to 414. After inserting the guidewire, mean flow rates were reduced to 54, 132, and 184 ml/min. Corresponding $\Delta \tilde{p}$ and $\Delta \tilde{p}$ are summarized in Table 2. The axial pressure drop profiles for above flow rates are plotted in Fig. 7B. The CPR values increased from 0.09 to 0.39 as Re_e _increased from 109 to 367; however, they were less than the corresponding values for 'before guidewire insertion'. Moreover, for all flow rates, the mean distal pressure recovered within a shorter axial distance as compared to 'before guidewire insertion'. *This signify the dominance of viscous pressure loss (*$\Delta {\tilde{p}}_{vis}$*) over momentum change-related pressure loss (*$\Delta {\tilde{p}}_{mom}$*) for moderate stenosis*.

**Table 2:** Mean Pressure drop and mean pressure recovery values before and during guidewire insertion

	Before guidewire insertion						
	
% area stenosis	Flow (*Q*)	pressure drop ($\Delta \tilde{p}$)	pressure recovery ($\Delta {\tilde{p}}_{r}$)	Pressure recovery coefficient (CPR)	Pressure recovery factor (*η*)	CDP_e_	Re_e_
65% area (moderate) stenosis	57	1.32	0.26	0.42	0.48	17	125
	136	5.77	1.67	0.48	0.55	13	298
	189	9.80	3.50	0.52	0.59	11	414
80% area (intermediate) stenosis	54	6.29	0.80	0.45	0.47	89	122
	82	12.46	2.10	0.52	0.54	77	195
	132	27.97	4.66	0.46	0.48	68	294
89% area (severe) stenosis	44	10.17	1.80	0.46	0.47	215	99
	74	24.79	4.04	0.38	0.38	190	164
	93	37.85	6.05	0.36	0.36	184	207
							

	During guidewire insertion						
	
% area stenosis	Flow (*Q*)	pressure drop ($\Delta \tilde{p}$)	pressure recovery ($\Delta {\tilde{p}}_{r}$)	Pressure recovery coefficient (CPR)	Pressure recovery factor (*η*)	CDP_e_	Re_e_

65% area (moderate) stenosis	54	1.62	0.05	0.09	0.11	22	109
	132	7.29	1.25	0.35	0.41	17	262
	184	12.41	2.76	0.39	0.45	15	367
80% area (intermediate) stenosis	51	7.93	0.62	0.35	0.36	125	101
	75	15.07	1.35	0.35	0.36	109	151
	121	32.18	4.07	0.41	0.42	90	244
89% area (severe) stenosis	34	10.90	1.41	0.46	0.46	372	68
	61	26.76	3.57	0.37	0.37	291	122
	81	39.44	5.06	0.30	0.31	247	161

#### Intermediate (80% area) stenosis

**Figure 8:**
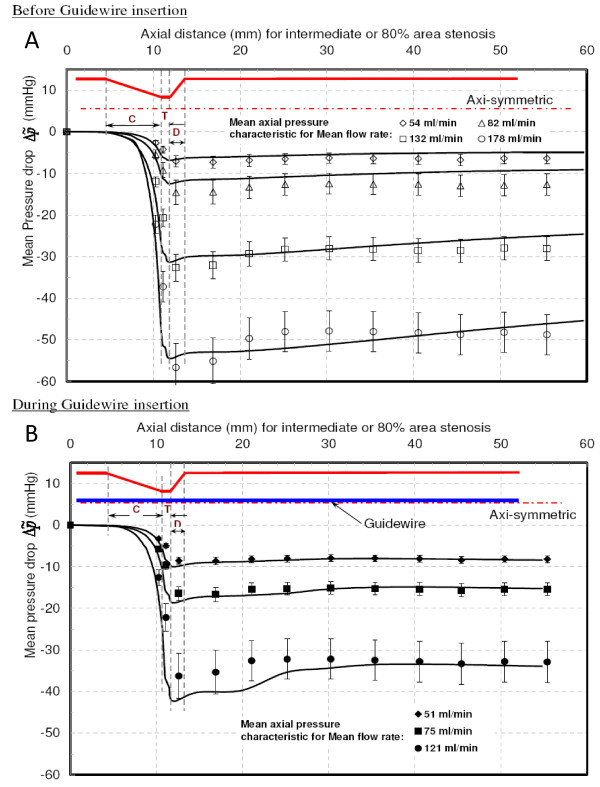
**Mean pressure drop vs. Axial distance for intermediate stenosis**. **A **– Before guidewire insertion. **B **– During guidewire insertion. C = converging region; T = throat region; D = diverging region. . . . .: Experimental data points. : Numerical results.

Before guidewire insertion (Fig. 8A), sudden pressure drop in the converging and throat region was observed with increased overall pressure drop as compared with moderate stenosis. The $\Delta \tilde{p}$ and $\Delta \tilde{p}$ are summarized in Table 2 for mean flow rates of 54, 82 and 132 ml/min. The CPR was nearly constant (0.46) for wide the range of Re_e _(122–294). Because of increased shear layer instabilities with flow separation and reattachment, the distal pressure was recovered with vortical cells formations [25,26]. The location of distal pressure recovery was approximately 14 mm downstream of the throat region for all flows. Generally, guidewire insertion adds viscous effects by increasing surface area and momentum-change effect by blocking more of the throat area. However, for intermediate stenosis, guidewire insertion relatively increased $\Delta {\tilde{p}}_{mom}$ by constricting more throat area than with $\Delta {\tilde{p}}_{vis}$. Table 2 summarizes the $\Delta \tilde{p}$ and $\Delta {\tilde{p}}_{r}$ and Fig. 8B shows the axial pressure drop profiles for 'during guidewire insertion condition'. The CPR was changed from 0.35 to 0.41 for Re_e _range of 101 to 150. The distal pressure recovery occurred approximately 13 mm distal to the diverging section. For larger mean flow rates, $\Delta {\tilde{p}}_{mom}$ was more dominant than $\Delta {\tilde{p}}_{vis}$, due to reduction in the throat area, as compared to moderate stenosis. The clinically measured fractional flow reserve (FFR) falls below the limiting value of 0.75 (leading to angioplasty or stent placement) [6] because of guidewire insertion; hence, true severity of intermediate stenosis is very difficult to diagnose.

#### Severe (89% area) stenosis

**Figure 9:**
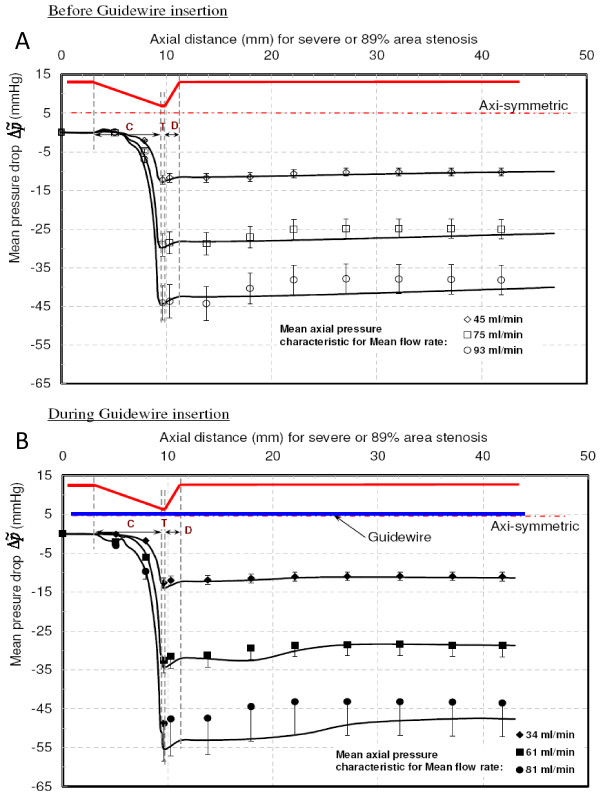
**Mean pressure drop vs. Axial distance for severe stenosis**. **A **– Before guidewire insertion. **B **– During guidewire insertion. C = converging region; T = throat region; D = diverging region. . . . .: Experimental data points. : Numerical results.

Before guidewire insertion, the overall pressure drop ($\Delta \tilde{p}$) was much higher with sharp pressure drop in the converging section as compared with that for moderate and intermediate stenosis (Fig. 9A), primarily because of an area constriction effect. The distal pressure recovery was highly unstable because of enhanced and organized vortical cells. The $\Delta \tilde{p}$ and $\Delta {\tilde{p}}_{r}$ are summarized in Table 2. The $\Delta \tilde{p}$ and $\Delta {\tilde{p}}_{r}$ were higher than those for moderate and intermediate stenosis for a similar Re_e _range. However, CPR was reduced from 0.46 to 0.36 for Re_e _range of 99–207. Before guidewire insertion, the vortical flow cells are much stronger than that during guidewire insertion. Figure 9B shows the axial pressure drop profile during guidewire insertion for 34, 61, and 81 ml/min. The guidewire insertion significantly reduced the blood flow with increase in $\Delta {\tilde{p}}_{mom}$. The CPR reduced from 0.46 to 0.30 for Re_e _range of 68–161. The pressure recovery values were observed at 17 and 13 mm distal to the stenosis before and during guidewire insertion, respectively. This type of significant stenosis is also known to cause ischemia in the subendocardium and coincides with symptomatic angina [13].

This study also suggests that the tip of the guidewire sensor should be positioned at sufficient distance distal to the stenosis. The pressure sensor tip should be positioned after the pressure recovery in order to avoid inaccuracy in distal pressure measurement. From axial pressure drop plots, shown in Figs. 7, 8 and 9, it is evident that the pressure tip should be at least 14 mm distal to the stenosis.

### Mean pressure drop and flow relation

**Figure 10:**
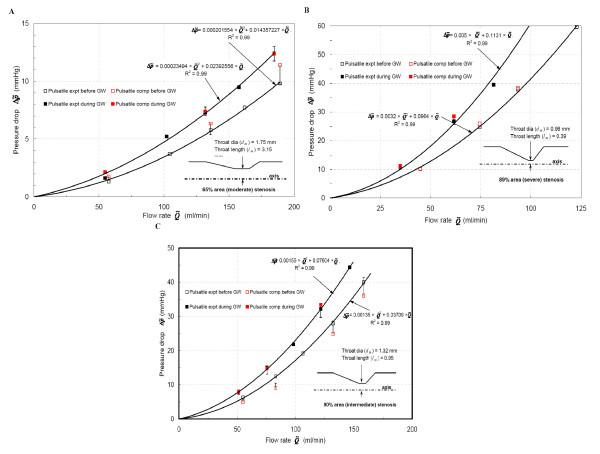
**Mean pressure drop vs. Mean flow rate**. **7A **– Moderate stenosis. **7B **– Intermediate stenosis. **7C **– Severe stenosis.

The time averaged transient pressure drop-flow ($\Delta \tilde{p}-\tilde{Q}$) characteristic for each stenosis geometry is compared and validated numerically (Fig. 10). The experimentally measured time averaged trans-stenotic pressure drop, pressure recovery, flow rate and Reynolds number are summarized in Table 2. The corresponding quadratic correlation, as provided in method section: formulation, is presented in Fig 10 for each stenosis geometry for pulsatile flow. Then the overall $\Delta {\tilde{p}}_{vis}$ and $\Delta {\tilde{p}}_{mom}$ for each stenosis model can be compared based on the coefficients 'A' and 'B'. These coefficients are summarized in Table 3.

**Table 3:** Viscous (A) and momentum-change (B) coefficients; Pulsatile flow values are obtained from Figs 7A, B, and 7C

Flow status	Steady flow				Pulsatile flow			
Stenosis severity	Before guidewire		During Guidewire		Before guidewire		During Guidewire	
	
	A	B	A	B	A	B	A	B

Moderate	1.46E-04	1.00E-02	1.87E-04	1.85E-02	2.01E-04	1.44E-02	2.34E-04	2.39E-02
Intermediate	4.70E-04	2.65E-02	8.00E-04	4.34E-02	1.35E-03	3.71E-02	1.55E-03	7.60E-02
Severe	2.50E-03	2.79E-02	4.00E-03	6.01E-02	3.20E-03	9.64E-02	5.00E-03	1.13E-01

For all stenotic models, the guidewire insertion increased the trans-stenotic pressure drop. The mean pressure drop for moderate stenosis (65% area stenosis) was governed by the viscous component, $\Delta {\tilde{p}}_{vis}$, which was further augmented by guidewire insertion (Fig. 10A; coefficient 'B' in Table 3). Due to dominance of $\Delta {\tilde{p}}_{mom}$, $\Delta \tilde{p}$ increased noticeably during guidewire insertion for severe stenosis (89% area stenosis) as shown in Fig. 10C. Before guidewire inserted condition, coefficient 'A' for severe stenosis increased by factors of 16 (= 32 × 10^-4^/2.01 × 10^-4^) and 2.4 (= 32 × 10^-4^/13.5 × 10^-4^), as shown in Table 3, from moderate and intermediate stenosis (80% area stenosis), respectively. During guidewire insertion, coefficient 'A' for severe stenosis increased by factors of 21 (= 50 × 10^-4^/2.34 × 10^-4^) and 3.2 (= 50 × 10^-4^/15.5 × 10^-4^), as shown in Table 3, from moderate and intermediate stenosis, respectively. *This confirms the dominance of *$\Delta {\tilde{p}}_{mom}$*for severe stenosis*. Interestingly, the intermediate stenosis was a trade-off point for which the dominance of $\Delta {\tilde{p}}_{vis}$ and $\Delta {\tilde{p}}_{mom}$ could not be clearly distinguished (Fig. 10B). *Thus, the guidewire insertion acts as an enhancer of viscous-losses in moderate stenosis, whereas it is the momentum-change pressure loss that dominates the severe stenosis and this distinction is unpredictable for intermediate stenosis*.

In addition, comparison of coefficients 'A' and 'B' for reference steady flow (not shown in the figure; shown in Table 3) and pulsatile flow showed that the pulsatile flow increased momentum-change effects significantly. For a selected mean flow rate, pressure drop for steady flow was lower than the time-averaged pulsatile flow cases. The comparison of steady and pulsatile flow coefficients shows that pulsatile flow increased $\Delta {\tilde{p}}_{mom}$ considerably than $\Delta {\tilde{p}}_{vis}$. *The pulsatile flow enhances momentum-change pressure loss due to the significant effect of convective term: *$\partial \bar{u}/\partial t$.

### Overall pressure drop coefficient (CDP_e_)

**Figure 11:**
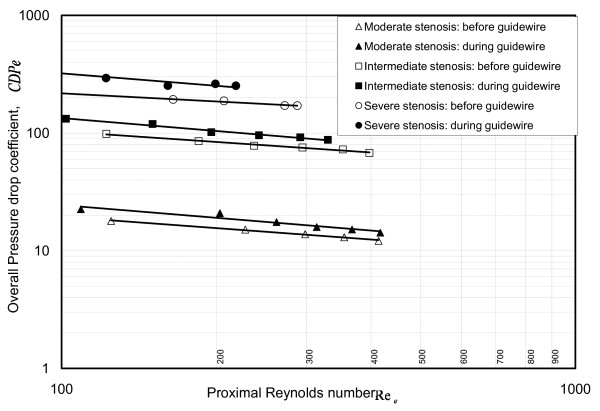
**CDP_e_-Re_*e *_correlations**.  represents pulsatile flow.

The CDP_e_, a non-dimensional quantity, represents the fluid flow resistance added by any arterial conduit having both momentum-change- and viscous- related pressure losses. The coronary stenosis adds resistance to flow by blocking more lumen area and its severity ranges from moderate stenosis offering negligible blood flow resistance to the severe stenosis contributing considerable blood flow resistance. Figure 11 shows mean CDP_e _vs. Re_e _characteristic for each stenosis and flow condition before and during guidewire insertion. The CDP_e _was primarily dependent on stenosis geometry, shape of flow pulse, presence of guidewire and flow rate. The mean CDP_e _increased from 17 ± 3.3 for moderate stenosis to 287 ± 52 for severe stenosis (n = 3, p < 0.01 using unpaired t-test). For a given stenosis, values of CDP_e_: decreased with increase in Re_e_; increased after guidewire insertion; and are higher for pulsatile flow than those for steady flow cases.

Before guidewire insertion, from basal to hyperemic flow, CDP_e _decreased from 215 to 184 for severe stenosis and from 17 to 12 for moderate stenosis. Similarly, during guidewire insertion, CDP_e _changed from 372 to 247 for severe stenosis and from 22 to15 for moderate stenosis. The statistically significant difference in the range of CDP_e _values between moderate and severe stenosis can be used in the clinical practice in diagnosing the severity of stenosis.

**Figure 12:**
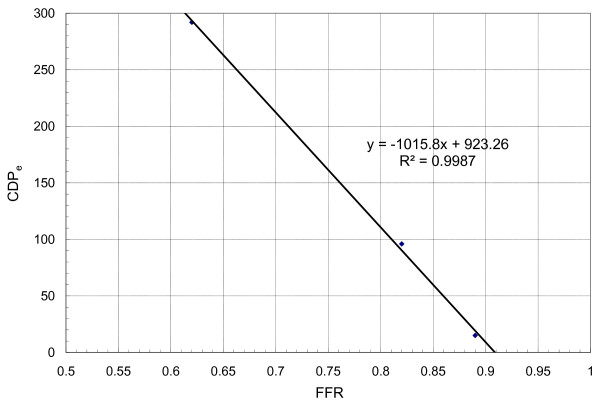
FFR-CDP_e _correlation showing a linear variation. The linear correlation indicates CDP_e _could be a viable diagnostic parameter under clinical setting.

Figure 12 shows FFR-CDP_e _correlation showing a linear variation. The CDP_e _increased by a factor of 15 from moderate to severe stenosis model. This wide range of CDP_e _can be used to delineate the severity of coronary stenosis. Unlike FFR, CDP_e _is not limited between small range; therefore a better and much accurate cut-off value can be established for CDP_e _after human clinical trials. The linear correlation indicates CDP_e _could be a viable diagnostic parameter under clinical setting.

### Pressure recovery factor (*η*)

Pressure recovery, downstream of the stenosis, can be quantified by the pressure recovery factor (*η*). For given flow rate, as stenotic severity increases from moderate to intermediate, the CPR increases, signifying increased pressure recovery, while approaching CPR_8_. As a result, *η *could also increase to a limiting value of unity. Similarly, for a particular stenosis, as flow increases, *η *increases due to increase in CPR. Table 2 compares the *η *values, calculated before and during guidewire insertion. From basal to hyperemic flow, during guidewire insertion, *η *increased from 0.11 to 0.45 for moderate stenosis and from 0.36 to 0.42 for intermediate stenosis. However, it decreased from 0.46 to 0.31 for severe stenosis. Similarly, before guidewire insertion, *η *increased from 0.48 to 0.59 for moderate stenosis, remained nearly constant (0.47 to 0.48) for intermediate stenosis and reduced from 0.47 to 0.36 for severe stenosis. This indicates that the guidewire insertion reduces the ability of the diverging section to recover the downstream pressure. In future, the CPR and *η *values can be obtained by guidewire pressure pullback profile in conjunction with the area measurement by diagnostic imaging techniques (e.g. IVUS, angiograph) in clinical settings.

## Discussion

The present study examines the pressure drop-flow relation for the range of epicardial coronary stenoses with quantification of $\Delta {\tilde{p}}_{vis}$ and $\Delta {\tilde{p}}_{mom}$ before and during guidewire insertion. More importantly, this research provides further hemodynamic insights on clinical data [13,28] and corroborates our earlier numerical studies [25,26]. The effect of guidewire eccentricity in relation to lumen diameter has also been performed, which is discussed at the end of this section.

The large difference between pressure drop values for the same mean flow implies that the contribution of viscous and momentum-change related pressure drops are different for *steady *and *pulsatile *flows. The pulsatile flows, having different $\partial \bar{u}/\partial t$ values, show diverse pressure drop values. Hence, it is necessary to distinguish the *steady *and *pulsatility *flow effects on viscous- and momentum-change pressure losses. Further, the effect of physiological parameters such as pulse shape, heart rate, and systole to diastole ratio are needed to be studied in the future.

Depending upon blood disorder, the effect of *blood viscosity *on diagnostic parameters should be analyzed since it may vary from patient to patient. The blood viscosity may affect the diagnostic procedure for intermediate and moderate stenosis as pressure drop for these geometries are dominated by viscous losses. In contrast, for the severe stenosis cases only momentum-change effects are significant and thus, blood viscosity may not have significant effect on diagnostic parameters.

A new diagnostic parameter '*lesion flow coefficient*' (LFC; see Additional file 2) was introduced to assess the severity of epicardial stenosis [15,17]. LFC is the ratio of square root of CDP_8 _(a limiting value of pressure drop coefficient at very high flow where viscous losses can be neglected over momentum-change losses) and square root of CDP_m _(pressure drop coefficient evaluated at the site of constriction at hyperemic flow where viscous and momentum-change pressure losses are equally important). As the flow increases, the CDP_m _approaches CDP_8_, leading to an LFC value of unity. The relation between CDP_m_, CDP_e_, CDP_8 _and LFC are summarized in Additional file 2. *CDP*_∞ _can be expressed as (1-1/*α*)^2 ^where, *α *is area ratio term obtained from equation 5 [17]. Thus, LFC is a function of flow rate, the severity of stenosis, guidewire size and the pulse shape. The nature of viscous- and momentum change-related pressure losses for CDP_e _will help in understanding the characteristic of the LFC. The detailed axial pressure drop profiles for each stenosis severity obtained from this study provide additional insights for determining viscous- and momentum change-related pressure losses.

A wide range of CDP_e _reveals that it can itself be used as a *diagnostic index *and thus, it has its own clinical importance. Since calculation of CDP_e _requires simultaneous pressure and flow measurement, it may be used to detect epicardial stenosis under presence of microvascular disease. The wide and non-overlapping range of CDP_e_, e.g. 14–22 for moderate stenosis, 87–132 for intermediate stenosis and 251–375 for severe stenosis may be useful for the estimation of severity of coronary stenosis. This *in-vitro *data will be helpful to design future pre-clinical and clinical trials.

The *distal pressure recovery *and *η *signifies the influence of pressure recovery, distal vascular-bed pressure and position of epicardial stenosis in relation to the location of guidewire on diagnostic parameters. The pull-back image obtained by IVUS catheter and pressure pull-back profile obtained by pressure guidewire can be recorded in a catheterization laboratory and may be combined to determine distal pressure recovery and *η*.

### Physiological limitations to the distal coronary pressure-flow data

**Figure 13:**
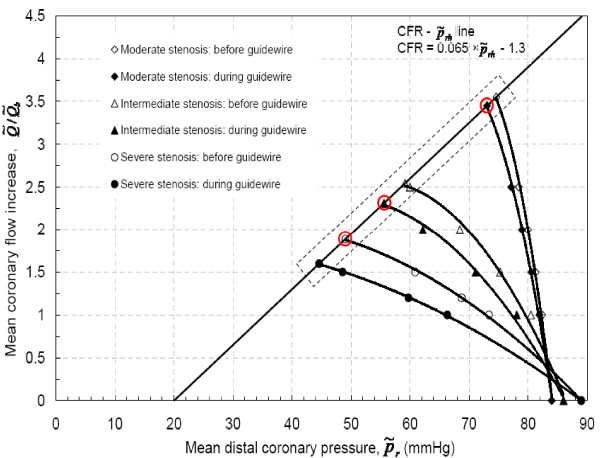
$CFR-{\tilde{p}}_{rh}$ line for mean pressure and flow data.

The $\Delta \tilde{p}-\tilde{Q}$ curves can be used to evaluate non-invasive diagnostic parameters [15]. The current *in-vitro *experimental data only simulate epicardial coronary hemodynamic and does not simulate the coronary microvasculature. The hyperemic flow range observed in human, governed by microvasculature, can be obtained by constructing coronary flow reserve vs. hyperemic distal recovered pressure ($CFR-{\tilde{p}}_{rh}$) characteristic (where subscript '*h*' indicates hyperemic flow). Therefore, the $CFR-{\tilde{p}}_{rh}$ line was constructed based on physiological limits, without microvascular impairment, reported in Table 4. Figure 13 shows that the intersection of $CFR-{\tilde{p}}_{rh}$ line and $\Delta {\tilde{p}}_{h}$ curves (for ${\tilde{p}}_{rh}$, $\tilde{Q}/{\tilde{Q}}_{b}-{\tilde{p}}_{rh}$ = CFR) for each stenosis, before and during guidewire insertion, are related by: $\tilde{Q}={\tilde{Q}}_{h}$, where ${\tilde{Q}}_{h}/{\tilde{Q}}_{b}$ is hyperemic flows and ${\tilde{p}}_{rh}={\tilde{p}}_{a}-A\times {\tilde{Q}}_{h}^{2}-B\times {\tilde{Q}}_{h}$. However, in clinical scenario, hemodynamic measurements are performed only by inserting guidewire or by invasive method. Consequently, hyperemic trans-stenotic pressure drop was estimated based on Fig. 13 for a given hyperemic flow for each stenosis severity. Further, estimated hyperemic pressure recovery, CDP_e _and *η *are provided in Table 5.

**Table 4:** Mean Hemodynamic end points in terms of CFR and ${\tilde{Q}}_{h}$

Stenosis	Coronary flow reserve (CFR)	Proximal pressure (${\tilde{p}}_{a}$) mmHg	Hyperemic trans-stenotic pressure drop ($\Delta \tilde{p}={\tilde{p}}_{rh}-{\tilde{p}}_{a}$) mmHg	Hyperemic distal recovered pressure (${\tilde{Q}}_{h}$) mmHg	Fractional flow reserve (FFR)
Moderate [13]	3.6 ± 0.3	84 ± 3	7.4	75.2	0.89
Intermediate [16]	3.3	86	14.3	70.4	0.82
Severe [13,28]	2.3 ± 0.1	89 ± 3	34	55	0.62

**Table 5:** Estimated hyperemic diagnostic parameters during guidewire insertion

% area stenosis	Estimated Hyperemic flow (from Fig. 10)	Estimated pressure drop (mmHg) $\Delta \tilde{p}=A\times {\tilde{Q}}^{2}+B\times \tilde{Q}$	Pressure recovery $\Delta {\tilde{p}}_{r}$ (mmHg)	Pressure recovery coefficient (CPR)	Pressure recovery factor (*η*)	CDP_e_
65% area (moderate) stenosis	172	11	2.5	0.41	0.46	15
80% area (Intermediate) stenosis	116	31	3.7	0.40	0.42	96
89% area (Severe) stenosis	80	45	5.0	0.31	0.31	292

### Eccentricity effect

**Figure 14:**
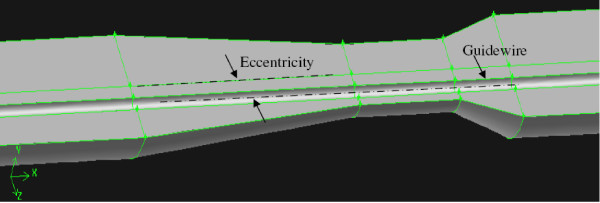
Three dimensional half plane-symmetric model showing eccentricity in moderate stenosis.

**Figure 15:**
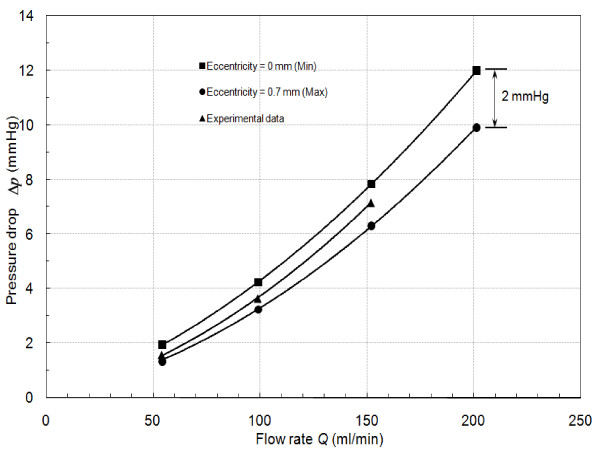
Comparison the steady state experimental pressure data with that obtained numerically for the extreme eccentricity conditions.

Maximum difference between the experimentally measured and numerically calculated $\Delta \tilde{p}$ was 20% during guidewire insertion. This difference is expected due to the assumption of concentric guidewire position in the numerical formulation. (The guidewire insertion was simulated by placing a guidewire of diameter 0.35 mm in concentric configuration with the stenosis axis as shown in Fig. 6A). In contrast, the guidewire was observed to lie eccentrically and fluctuates with the pulsatile flow inside the stenotic test section during the experiments. Thus, position of guidewire inside the stenosis test section was unpredictable. The effect of guidewire eccentricity was well explained in the literatures for straight artery under pulsatile flow [29] and under steady flow [30]. To validate the eccentricity effect in moderate stenosis, 3 dimensional half plane-symmetric models were constructed (Figure 14). The extreme conditions of guidewire placements namely, concentric and maximum eccentric positions were simulated and steady state pressure drop calculations were performed using the procedure explained in the method section. Figure 15 compares the steady state experimental pressure data with that obtained numerically for these extreme conditions. The pressure drops between above stated extreme conditions differ by ~2 mmHg which matches closely with previously reported numerical data [30]. The eccentricity effect under pulsatile flow condition should be quantified in future.

## Conclusion

The guidewire insertion caused reduction in hyperemic flow and increased the trans-stenotic pressure drop, that affected the viscous- and momentum change-related pressure losses. The pressure drop coefficient (CDPe) increased by a factor of 15 from moderate to severe stenosis model and thus diminishes the overall effect of guidewire flow obstruction and related misdiagnosis during invasive functional measurements. This wide range of CDPe can be used to delineate the severity of coronary stenosis. Unlike FFR, CDPe is not limited between small range; therefore a better and much accurate cut-off value can be established after human clinical trials. The pressure recovery factor (*η*) quantifies the recovered distal vascular-bed pressure downstream of the stenosis. The geometrical details of coronary stenosis included in the expression for *η *combines the functional and anatomical information. CDPe and *η *need to be further evaluated for pre-clinical studies in order to distinguish between epicardial and microvascular dysfunction.

## List of Abbreviations used

*A *– flow cross-sectional area, *d *– diameter of the vessel at specified section, *l *– length of vessel at specified section, *p *– vessel pressure, Δ*p *– pressure drop (pressure gradient in clinical literature): Δ*p*_*c *_+ Δ*p*_*m *_– Δ*p*_*r*_, Δ*p*_*r *_– distal pressure recovery: ${\tilde{p}}_{r}-{\tilde{p}}_{m}$, Δ*p*_*m *_– pressure drop in throat region, Δ*p*_*c *_– pressure drop in converging section, *Q *– volumetric flow rate (ml/min); *r *– vessel radius: *d*/2, Re – proximal Reynolds number: 4$\tilde{\text{Q}}$/[*πνd*_*e*_(1 + *d*_*i*_/*d*_*e*_)], *t *– time, *u *– axial velocity, *u*_*i *_– velocity vector, *u*_*r *_– radial velocity vector, *α *– Area ratio term, $\dot{\gamma }$ – shear rate, *λ *– time constant in non-Newtonian, Carreau model, *ν *– kinematic viscosity: *μ*/*ρ*, *ρ *– blood density, *μ *– dynamic viscosity, T – period of the cardiac cycle.

Subscripts

*a *– aorta, *c *– converging section of the stenosis, *e *– proximal section to the stenosis, *h *– hyperemic condition for flow and pressure, *i *– guidewire, *m *– *t *– minimum temporal value, *m *– throat section, *o *– mean diameter, *p *– *t *– peak temporal value, *r *– distal section/diverging region of the stenosis.

Superscripts

^~ ^– time average (mean) quantity over cardiac cycle, ^- ^– spatial average quantity across the vessel

## Competing interests

The authors declare that they have no competing interests.

## Authors' contributions

KDA carried out the stenotic models and experimental setup design, trans-stenotic pressure-flow measurements, numerical calculations and participated in the sequence alignment and drafted initial versions of the manuscript under supervision of RKB. SFK provided the important clinical pressure-flow measurement information. TAH and MAE provided clinical insights of the guidewire diagnostics and formatted the background section. LHB provided technical insights into this research during experimental setup, stenosis model design, experimental and numerical data analysis and critically revised the draft. All authors read and approved the final manuscript.

## Supplementary Material

Additional File 1 Appendix – I: Effect of blood viscosity. This is the appendix for the manuscript explaining the effect of blood analog fluid viscosity on the trans-stenotic pressure drop ($\Delta \tilde{p}$). Click here for file

Additional File 2 Appendix – II: Relation between cardiovascular diagonostic parameters. This is the appendix for the manuscript explaining the relation between cardiovascular diagonostic parameters: CDP_e_, CDP_m _and LFC. Click here for file
