# Supplementary material for: Hemodynamic diagnostics of epicardial coronary stenoses: in-vitro experimental and computational study
Source: Biomed Eng Online. 2008 Aug 27;7:24. doi: 10.1186/1475-925X-7-24 (PMC2556321; doi:10.1186/1475-925X-7-24)
Supplement: Additional File 1 — Appendix – I: Effect of blood viscosity. This is the appendix for the manuscript explaining the effect of blood analog fluid viscosity on the trans-stenotic pressure drop (Δp˜). [file 1475-925X-7-24-S1.doc]

# Appendix I: Effect of BAF viscosity on pressure drop ()

The viscosity of the blood analog fluid is less than the reference blood viscosity in a mid shear rate region as shown in Fig. 5. Hence, it is important to check the effect of change in blood viscosity on the trans-stenotic pressure drop for the same flow pulse. The numerical calculations were carried out with viscosity observed for real blood[24], before and during guidewire insertion for all stenosis models[25, 26]. The difference between the pressure drop for blood analog fluid and that for real blood viscosity were insignificant (maximum difference of 5%).
