# Supplementary material for: Hemodynamic diagnostics of epicardial coronary stenoses: in-vitro experimental and computational study
Source: Biomed Eng Online. 2008 Aug 27;7:24. doi: 10.1186/1475-925X-7-24 (PMC2556321; doi:10.1186/1475-925X-7-24)
Supplement: Additional File 2 — Appendix – II: Relation between cardiovascular diagonostic parameters. This is the appendix for the manuscript explaining the relation between cardiovascular diagonostic parameters: CDPe, CDPm and LFC. [file 1475-925X-7-24-S2.doc]

# Appendix-II: Relation between CDPe, CDPm and LFC

In our recent publications [15, 17], we have reported for the first time a cardiovascular diagnostic parameter, lesion flow coefficient (LFC), that combines the functional endpoints with geometric or anatomical endpoints.  The hemodynamic endpoints include the throat pressure drop coefficient (CDP_m_), whereas the geometric endpoints include the ratio of a stenosis minimal cross-sectional area and the area of the native artery. Hence, LFC is defined as

(1)

where, throat pressure drop coefficient (CDP_m_) is the ratio of overall pressure drop (, superscript ‘~’ indicates temporal average quantity) to the proximal dynamic pressure (), i.e.,

(2)

where, is blood density; is spatial and temporal mean blood velocity at the minimum vessel area (superscript ‘–’ indicates spatially average quantity). The quantity CDP_∞_ represents CDP_m_ at very high Reynolds number (Re) flow, i.e.,

(3)

where, α is area ratio before guidewire insertion and α_i_ is area term during guidewire insertion. The throat pressure drop coefficient (CDP_m_) and proximal pressure drop coefficient (CDP_e_) are related by mass balance equation across the proximal and throat section of the stenosis and is as follows:

i.e. (4)

Thus, results of CDP_e_-Re_e_ characteristic (Fig. 11) from this experimental research may be useful for the characterization and further development of CDP_m_ and LFC.
